# The impact of the city skyline on pleasantness; state of the art and a case study

**DOI:** 10.1016/j.heliyon.2021.e07009

**Published:** 2021-05-08

**Authors:** Mehrdad Karimimoshaver, Mastooreh Parsamanesh, Farshid Aram, Amir Mosavi

**Affiliations:** a Department of Architecture, Bu-Ali Sina University, Hamedan, Iran; b Escuela Técnica Superior de Arquitectura, Universidad Politécnica de Madrid-UPM, Madrid, Spain; c Neumann Faculty of Informatics, Obuda University, 1034, Budapest, Hungary; d Department of Informatics, J. Selye University, 94501, Komarno, Slovakia

**Keywords:** Skyline, Aesthetics, Pleasant, Landscape, Urban design, Sustainable development

## Abstract

The research deals with the effect of skylines on citizens' pleasantness. The research method is based on the respondents' judgment of the color images of the skylines. 360 citizens were asked to complete a questionnaire to express their opinions and preferences along with the reasons. Three types of nature, traditional, and contemporary skylines were identified as the dominant skylines. The results showed that people prefer the nature and the traditional skyline over the contemporary skyline. They introduced some features as peacefulness, memorability, and distinctiveness as the reasons for their choice. The people's residence place could influence their attitudes toward the skyline, and most of those living in the areas with contemporary contexts selected the skyline of their contemporary context as the favorite skyline. They did not look for the sense of peacefulness in the skyline, but they underlined attractiveness. Variables of age and gender had no effect on the preferences; however, by an increase in education level, the tendency to select the traditional and contemporary skyline increased.

## 1 Introduction

### 1.1 The city skyline

Our houses are usually decorated with many photos and paintings to convey personal messages to others regarding personal interests and the flavour of the rooms. The world cities are very similar to the rooms, in the sense that they have different functions and flavours, and to convey the messages, the images are needed to be used. If we imagine the city as a room, the skylines are the images and tableaus mounted on the walls (Booth, 2012).

The skylines are the outline of buildings or other objects with the sky's background (Hornby et al., 1982). The skyline plays an important role in the beauty, meaning and urban views (Gholami et al., 2019; Karimimoshaver and Winkemann, 2018; Karimimoshaver et al., 2015; Guney et al., 2012). The term “urban skyline” refers to the characteristics of the buildings that make up the natural scenes during the day and shadow during the night (Lim and Heath, 1994).

One of the most important buildings in the skyline is tall buildings (see Karimimoshaver et al., 2020a, Karimimoshaver et al., 2020b). In the literature, any building that is significantly taller than other buildings is considered a tall building. The Council on Tall Buildings and Urban Habitat (CTBUH) developed the defining tall buildings in three category including height relative to context, proportion, and technologies relevant to tall buildings ("Council on Tall Buildings and Urban Habitat", 2021) which shows the importance of tall buildings in the city. Although in many countries the height of tall buildings is 20–30 m, these numbers vary in different contexts (see Karimimoshaver and Winkemann, 2018). Another importance of tall buildings is that many tall buildings are newer buildings than other buildings and can be controlled to some extent to control the skyline. Anyway, what matters is that tall buildings significantly impact the city skyline (Tavernor and Gassner, 2010; Samavatekbatan et al., 2016). After the widespread revolts against tall buildings in the 1970s, their construction was limited not only in Asia but also in Europe and America. However, in the throes of the election controversies in London, the mayor of London, highlighted the advantages of tall buildings and their iconic roles, and supported their expansion in improving the quality and beauty of London. Subsequently, considerable changes happened in the buildings designed in London, to the extent that it was announced that, up to 2015, the cities could have 18 to 20 skyscrapers. Nowadays, the aesthetic dimension of tall buildings and their impact on the urban skyline, especially for the cities that intend to upgrade their universal positions (e.g., Sydney and Kuala Lumpur), is inevitable. This has led to the increased attention paid to iconic architectures. For example, some monument such as the Guggenheim Museum in Bilbao has become a cultural symbol and alerted the city's face and created tourism (Charney, 2007).

Many cities have focused on their image as a potential tourist destination (Masoud et al., 2018; Ruiz et al., 2019). A review of tourist travelogues, the postcards manufactured for sale to tourists, and the use of the images on the TV indicate the fact that how the form of the skyline is the most essential part of the city image. In most cases, tall buildings are the most dramatic city elements (Rød and van der Meer, 2009; Short, 2007). By recognizing the increasing effects of tall buildings on the aesthetic quality of urban environments, a tendency was generated to formulate regulations that consider aesthetic impact as the most important and obvious objective (Delafons, 1990; Short, 2012).

There is limited literature on the urban skyline. The research carried out by Nasar (1990) indicates the scope of the research by the scholars and practitioners on bio-environmental aesthetics. The research by Appleyard (1979) and Appleyard and Fishman (1977) on how and why the buildings become the landmarks have remained for many years as the sole experimental research. Other researchers have sought to determine the degree of complexity and priority related to the buildings. Nasar and Terzano (2010) in another research conducted extensive studies on the skyline. In three studies, they used color photos to compare the natural scenes of the city skyline after dark and during the day. In the first study, the participants were asked to announce the rate of the pleasantness of each scene. They found out that the night skyline and natural scenes are more pleasing than the skyline during the day. The second research was carried out on a sample of 56 participants who were asked to select one favorite scene out of three sets of seven scenes as a frame to hang on the wall. The respondents were also asked to declare the reasons for the choice at the end. The most frequent scenes selected by the participants were the night skyline, then the nature skyline, and finally the day skyline. In the third research, the ratings of the selected standard features of each scene were obtained. The participants expressed natural scenes as more natural, orderly, open, and curvilinear, less complex, and as having smoother transitions than either day or night skylines; and they also expressed the night skylines as higher in complexity but lower in order than day skylines. The results indicate that preference for the categorizing of the natural scenes may depend on a combination of formal and content features (Nasar and Terzano, 2010).

Stamps (1991) examined the effect of height, complexity, and architectural style on the preference of buildings. By matching the order underlying the fractal geometry to the placement pattern of tall buildings, and creating several skylines by the same method, he collected responses and concluded that compliance with fractal geometry in the skyline brings about less satisfaction (Stamps, 2002).

Lim and Heath (1994) formulated a mathematical model that made possible the planning of the synthetic skylines with the characteristics that reflect those of real cities. Using this model, Smith et al. (1995) examined the interaction of the proportion and spacing of tall buildings in influencing preferences (Heath et al., 2000).

In another research by Zacharias (1999), the relationship between buildings and natural scenes was examined. He presented a comparative analysis of the preferences for relationships between buildings and landscapes. In the research, 11 scenes of tall buildings against a mountain ridge were designed. In another set, by raising buildings' height and height variation, the buildings were still below the mountain ridge. After collecting the respondents’ views on the preferences, it was indicated that the variation in building height but with all buildings below the mountain ridge accounted for the highest rate preferences.

In Shanghai, the way to show economic progress was the development of skyscrapers. These giant buildings are so appealing because they are related to modernization (Schilbach, 2016). However, European cities are still reluctant to build tall buildings (Oueslati et al., 2015). Europe does not like skyscrapers, and there are negative views on skyscrapers. Europeans believe that such buildings have led to the destruction of life and the cultural heritage of old European cities (McWhinnie et al., 2006). The historical context of a city is considered a historical asset. The preservation of such contexts is the most important reason to refuse the construction of such buildings (Koefoed, 2013).

### 1.2 Aesthetics and pleasantness

The term aesthetics derived from the Greek term Aisthetikos means sensory perception, and Aistheta means something sensually perceived. The term was first used by Alexander Baumgarten in the eighteenth century in the sense of recognition through the senses, i.e. sensory knowledge. Baumgarten applied the term the perception of beauty by the senses, especially in the field of art (Goldman, 1995).

There are several theories about aesthetics (see Karimimoshaver, 2013), and the historical background of the term indicates that there is no a single definition. In the following, only the terms more relevant to the subject of the current research are examined.

#### 1.2.1 Sensory aesthetics and sense of pleasantness

Sensory values result from pleasurable sensations such as touching, smelling, tasting, hearing, and observation. Santayana (1896) believes that while sensual pleasure is part of beauty, its evocative images are a part of the nature of the objects. Lang (1987) believes that energy stimuli such as light, color, sound, smelling, and touching are delightful architectural spaces for the user and observer. He states that very little is written about the aesthetic sensation and the existing literature is also heavily reliant on personal views. In certain situations such as passing through silhouette and bright areas, standing on the beach, feeling the wind blowing, breathing ozone-rich air, and a cool breeze blowing offshore at the time of heat, man becomes aware of aspects of sensory perception.

Canter and Stringer (1976) examine the interaction between the different senses of the human and the environment. They present an explanation and quantification of the sensory qualities of the environment and expression of the optimal levels of energy sources on the environment. The environment is examined in terms of thermal, acoustic, and visual factors, and the results are presented to understand the qualities of the architectural environment, city, and nature. If we believe in the idea proposed by Lang (1987) on the equivalence of the environment's beauty and its pleasantness, we can likely use quantitative indicators proposed by Canter and Stringer as standards for sensory aesthetics.

The most ancient answer about the origin of the beauty or artistic nature of a work is: art is a source of pleasure or enjoyment, and in support of this claim, it seems that most people who want to present a favorable comment on a book or film, they say “enjoyed” the book or the movie (Graham, 1997). Some philosophers have thought that the value of art is necessarily related to a sense of pleasure or enjoyment. Therefore, saying that a work is good is tantamount to saying that the work is enjoyable or pleasant (Gaut and Lopes, 2001).

#### 1.2.2 Aesthetics in built environment

Along with the set of definitions and procedural issues presented in the previous sections, it should be noted that a significant part of the urban design debates revolves around substantive issues including aesthetics. Golkar (1999a) points out that although urban design emerged from an aesthetic issue (urban beautification movement), it gradually covered many other important aspects such as functional, behavioral, and environmental considerations. However, the model presented by Golkar (1999b) on the components of quality of the urban design is introduced in the form of the three relevant components (functional, behavioral, and environmental), and is indicative of the strong role played by urban design in aesthetic debates.

Carmona (2003) believes that urban design has six dimensions including morphological, perceptual, social, visual, and functional dimensions, which the visual dimension considers physical aesthetic aspects of design. However the notion of Santayana on different types of environmental aesthetics must be taken into account. Based on his statement, aesthetics can be divided into three forms including sensory, physical, and symbolic (Santayana 1896, quoted by Lang 1987). Lang believes that Santayana's classification is still valid to organize the views on environmental design in general (and urban design in particular).

### 1.3 Research question

Despite existing studies on the skyline, it seems that the subject of the city skyline, especially its aesthetic dimension, has been neglected. Besides, among the few criteria proposed to restrict new building construction, there is little research that can measure people's preferences and interests. Therefore, this research attempts to examine what kind of skyline is favored by people and what would be the justifications on such choice. In other words, this research examines the sensory aesthetics and the impact of different types of skylines on citizens' sense of pleasantness.

## 2 Materials and methods

This research used the methods and techniques developed by Nasar and Terzano (2010) to evaluate the effect of aesthetic characteristics on people's preferences for the skyline. The assumption was that people desire for nature is a natural tendency. This tendency will increase their preference for nature skyline. In the meantime, it seems that among the categories including the skyline of the traditional context and that of the contemporary context, the former is more preferred due to people's natural preference toward sensory aesthetic features.

### 2.1 Case study: Kermanshah city

Kermanshah city, the capital of Kermanshah province, with an area of 8796 square kilometers, is located in the valley of Qarahsoo and its altitude is 1410 m above sea level.

This city is a combination of two main parts: 1- Traditional neighborhoods: with a discrete body and sometimes semi-ruined, 2- Contemporary neighborhoods: without a known architecture style, the texture of which is devoid of physical-spatial thought, as well as marginal settlements and with an uncontrolled texture.

The population of Kermanshah city according to the 2006 census is 497763 people. The statistical population in this study were shopkeepers and sellers who, in addition to having enough time and desire to complete the questionnaire, also had a suitable place to complete the questionnaire. Since the number of registered shopkeepers, between 18-60 years old, is 5710, based on Cochran's formula and with a 5% error rate, 360 questionnaires were completed by the participants. In this way, among the eight districts of Kermanshah, each of which has eleven neighborhoods, two neighborhoods from each district were randomly selected. After explaining that this is just a questionnaire for a scientific study, a survey was conducted of those who agreed to cooperate.

### 2.2 Questionnaire

The respondents were asked to observe photos of nature, traditional and contemporary skylines, and then answer the questions. The photos were presented in three nine-fold sets, and the extent of the subjects’ pleasantness was ranked. Then, out of three existing photo sets, the participants were asked to select one photo set as a frame for their home or workplace and present justifications for their selection.

The respondents' attitude during showing different sets of photos, based on the skyline theme and evaluating their opinions as a 7-point differential scale, was measured including artificial-natural, rectilinear-curvilinear, closed-open, abrupt-smooth transition, simple-complex, chaotic-orderly, regular-irregular (Table 1).

**Table 1:** Respondents' attitudes toward the visual features of skyline photos as a 7-point differential scale.

	Very high	high	relatively high	medium	relatively high	high	Very high	
Natural								Artificial
Curvilinear								Rectilinear
Open								Closed
Complex								Simple
Smooth transitions								Abrupt transitions
Orderly								Chaotic
Irregular								Regular

Respondents were also asked to record their district of residence on the map provided to them to examine the relationship between the skyline of the residence and the choice of the preferred skyline.

### 2.3 Classification of skyline types in the case study city

Many cities in Iran have a historical core around which new buildings have been built, which are physically different from the historic core. There are also natural and mountainous landscapes around many cities in Iran. These conditions have made it possible to see three types of the skyline in many cities of Iran and the case study city: traditional, contemporary and nature skylines.

Most traditional buildings have been built by experimental architects, which are the product of life experience and structural constraints, most of which are two-story buildings. Regarding the new buildings, it is necessary to mention that these buildings were built according to government regulations, but by the free market, most of which are 7-story buildings that are considered tall buildings in the case study city.

### 2.4 Photos

Photos used to survey people's opinions can be manipulated to control the variable or unmanipulated to prevent them from looking made-up. We preferred to avoid the first method because the changes were artificial and did the research based on original photos to ensure that the effect of the photos was closer to reality. Of course, this method also has disadvantages that were tried to reduce them as much as possible with the large number of photos that were examined to select the final photos for analysis and presentation to the respondents.

To achieve the same conditions underlying the all photos, the photos selected for the research had the following characteristics:

1 The shooting angle was neither very limited, nor very wide so that the variations in the skyline were clearly visible.
2 The skylines were selected from different parts of the city in order to display the mountainous landscape of the city as a geographical feature. Besides, to create similarities between the photos, an attempt was made to include a part of natural elements to all photos, such as trees in each photo.
3 Each of the human-made skylines reflected a particular period: traditional and contemporary.
4 Each skyline reflected a different form: relatively flat, steep, symmetrical, the buildings with rhythmic, long, and short distances, and the skylines reliant or non-reliant upon civil landmarks, minaret, dome, etc.
5 Due to the same conditions underlying the light and shadow, the photos were taken at a similar temporal situation and at 10 am and by the same lens.
6 Each skyline was required to be seen from a popular spot at various intervals (from the city's landmarks such as entrances, parks, and high points of the city, see Karimimoshaver et al., 2020). The main attempt was to take the photos at the same height from the ground.

**Figure 1:**
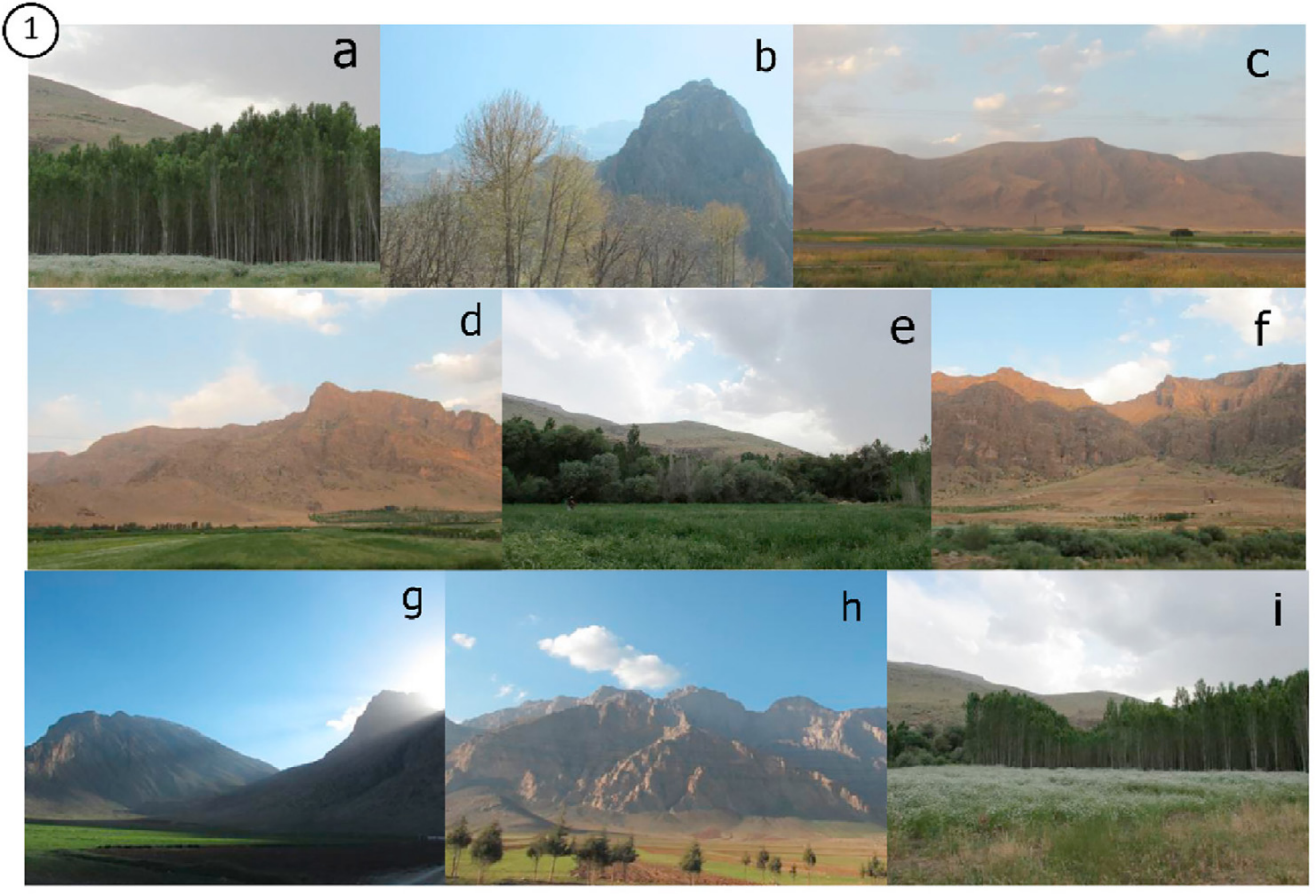
The final selected skylines as Nature skyline of Kermanshah city.

**Figure 2:**
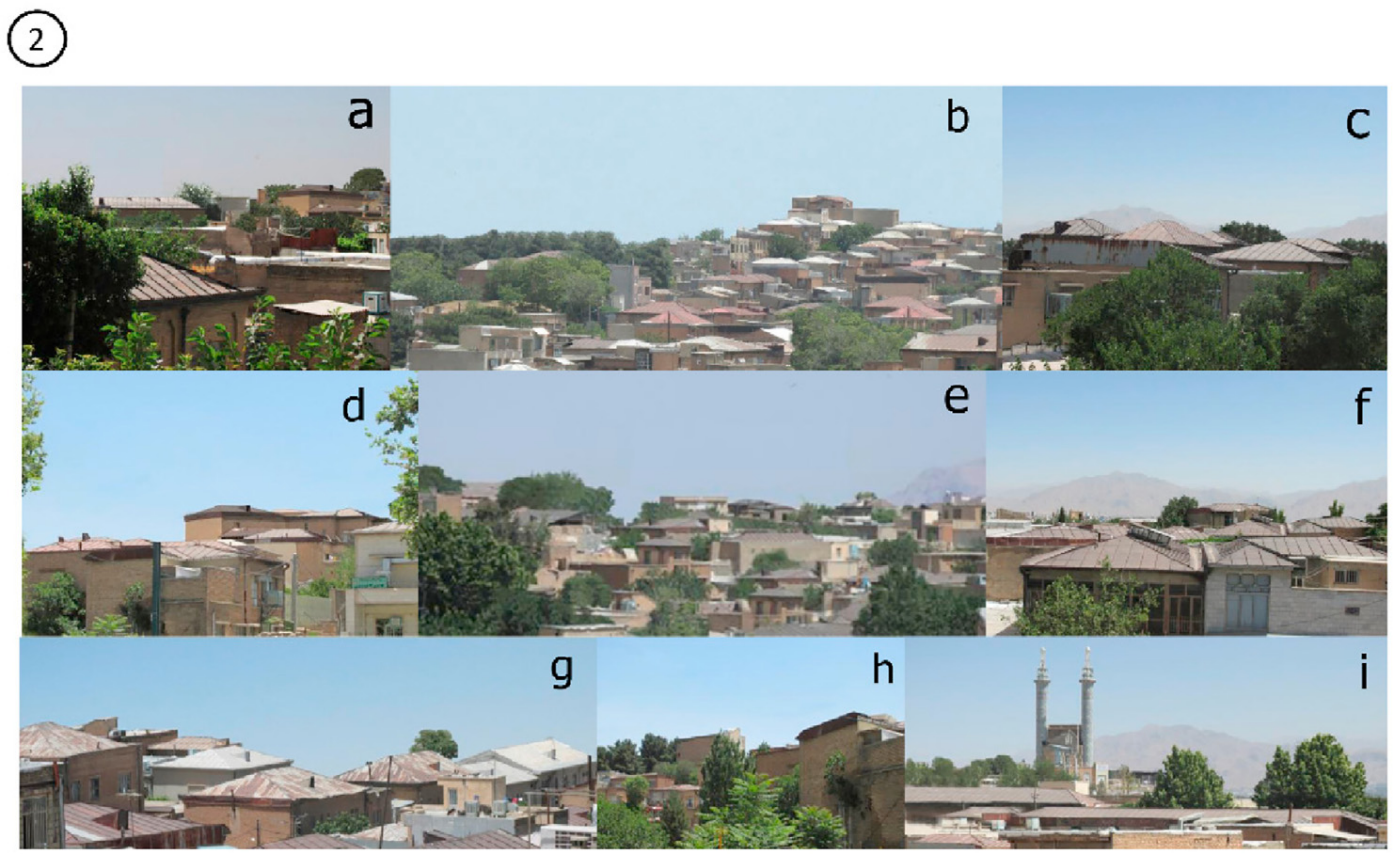
The final selected skylines as Traditional Skyline of Kermanshah city.

**Figure 3:**
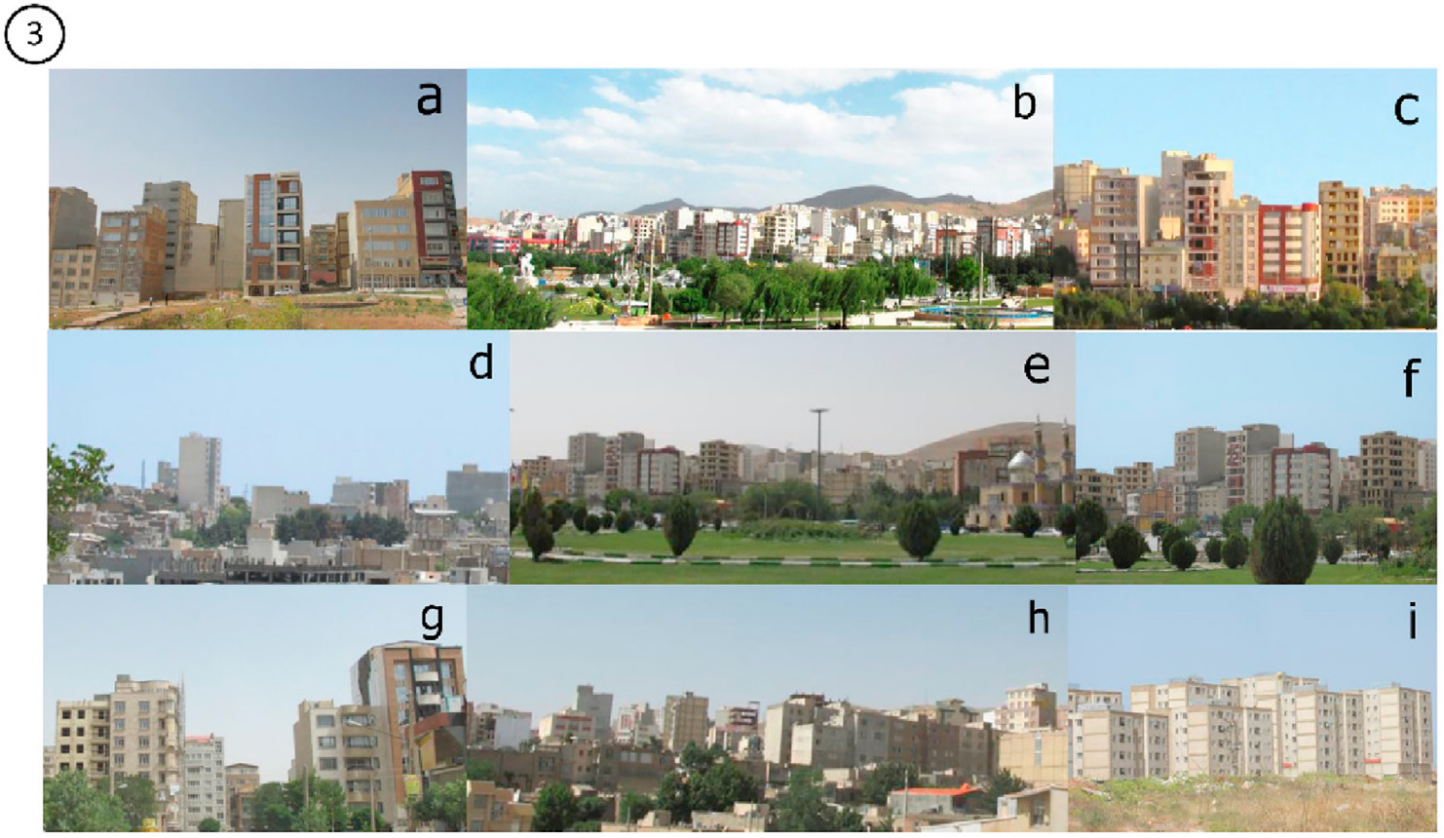
The final selected skylines as Contemporary Skyline of Kermanshah city.

**Figure 4:**
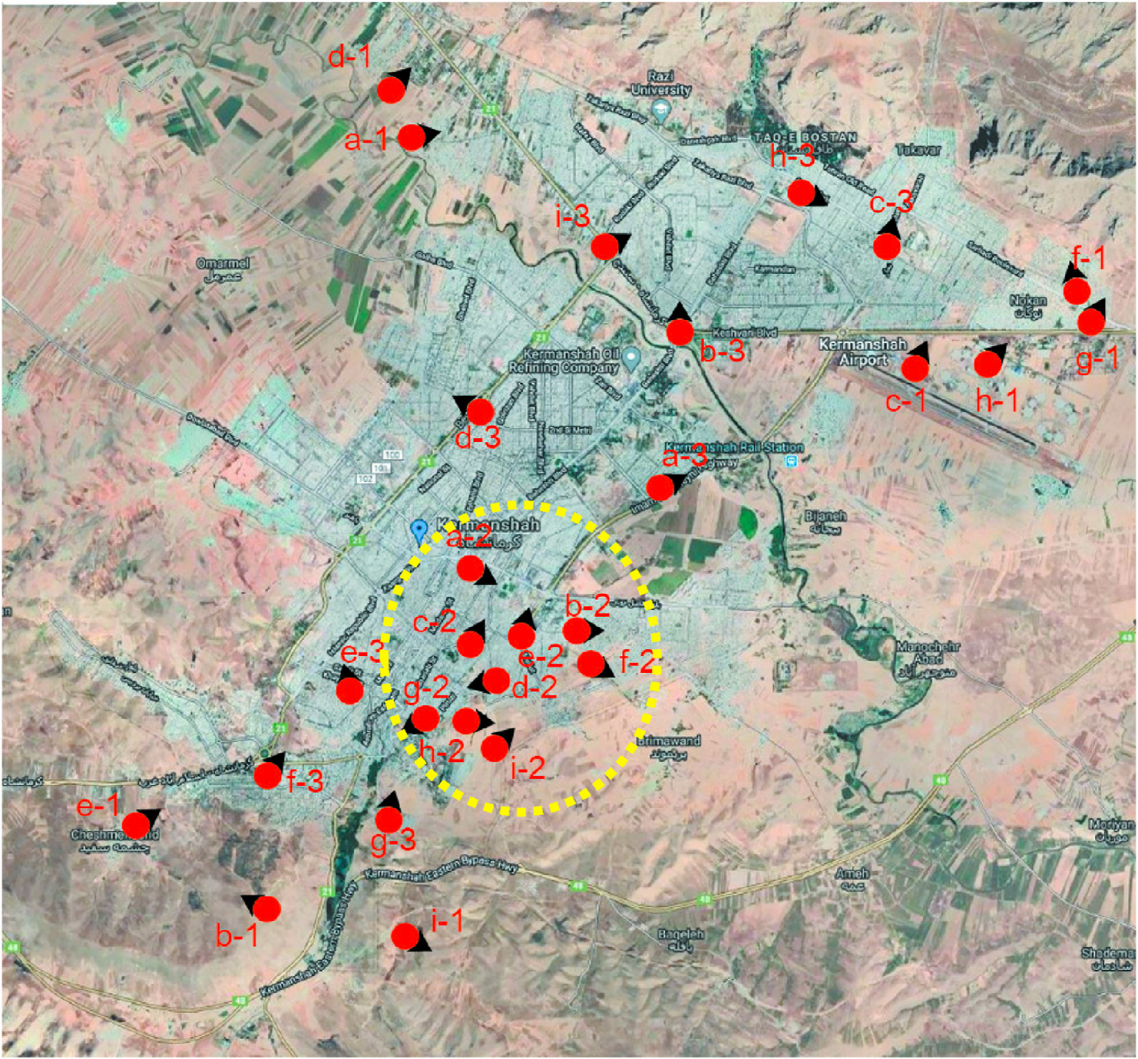
Satellite map of Kermanshah city (Source: Google Map, accessed 25 December 2020) as well as the location and direction of shooting photos in Figures 1, 2, and 3 (the traditional part of the city, which is related to Figure 2, is shown with a yellow dashed line).

Finally, out of 2000 photos taken from Kermanshah, three nine-fold sets of photos were selected (Figures 1, 2, and 3). The location and direction of the photo shooting and the traditional part of the city are shown in Figure 4.

The research methodology described above had been confirmed and followed the Obuda University's ethics guideline. This research does not contain any studies with human participants or animals performed by any of the authors.

## 3 Results

### 3.1 Descriptive analysis of the variables

Of the 360 respondents, more than 42.5% were female and 57.5 % were male. Regarding the age distribution of the respondents, 20 % of them were in the age range of 18–27 years, and 50 % were between 28 and 37 years old, 17.5% were between 38 and 47 years old, and 10.8% were 48–57 years old. Based on these findings, only 1.7% (n = 6) were over 57 years old. The average age of the respondents was 34 years and ten months. Of the total number of the subjects, 3 patients (0.8%) did not specify their education level. While among the people who specified their education level, 3.4% had a high school diploma and 21.8 % had a high school degree. Those with associate degree accounted for 14.3% of the respondents, while 37% had a bachelor's degree, and 18.5% had a master's degree. Finally, only 5% of the respondents had a doctoral education.

### 3.2 Ranking of the respondents’ pleasantness

The respondents' pleasantness toward the three sets of the photos was compared. To test the hypothesis of the difference in the respondents’ view compared to the three photo sets, Friedman test was used (Table 2).

**Table 2:** The respondents’ sense of pleasantness toward three skyline photo sets.

Photo Set		Mean Rank		Rank	
Nature skyline Photos		2.65		1	
Traditional Skyline Photos		1.90		2	
Contemporary Skyline Photos		1.46		3	
N: 360	Chi-Square: 295.379		df: 2		Asymp. Sig.: 0.000

Table 2 shows Friedman test result to rank the respondents' pleasantness toward three sets of the photos. Based on the test results, p-value is 0.000 and chi-square value at a confidence level of 99.9%, is significant. Therefore, the null hypothesis indicating the equal ranking of the three photo sets is rejected, the main assumption is confirmed, and the results can be generalized to the entire population of Kermanshah city. Therefore, the respondents’ pleasantness toward three photo sets had different rankings, which based on Table 2 pleasantness toward the photos of the nature skyline is ranked first and has accounted for the maximum value. Then, pleasantness toward the skyline photos of the traditional context is ranked, and finally pleasantness toward the skyline photos of the contemporary context.

### 3.3 Comparison between the respondents’ selection of three sets of photos to hang on the walls

The respondents were asked to select one of the photos to hang on the walls of the office or home. The null hypothesis is that all sets are selected to an equal extent and there is no difference between the selections of the sets. Therefore, in order to test this hypothesis, chi-square test is used (Table 3):

**Table 3:** Chi-square test of the comparison between the selections of the three photo sets for hanging.

Photo Set	Expected N		Observed N		Residual	
Nature skyline Photos	120.0		300		180.0	
Traditional Skyline Photos	120.0		48		-72.0	
Contemporary Skyline Photos	120.0		12		-108.0	
Total	360		360		0	
	N: 360	Chi-Square: 410.400		df: 2		Asymp. Sig.: 0.000

According to the results, the p-value was equal to 0.000; thus, the chi-square value was significant, and the main hypothesis (the difference in the selection of three sets of the photos for hanging on the wall) was confirmed, and the expected and observed frequencies were not the same. The most favorite skyline photos selected by the respondents include the nature skyline, the skyline of the traditional context, and finally the skyline of the contemporary context, respectively.

### 3.4 Comparison of the residence place and the selection of three sets of the photos to hang on the wall

One of the objectives of the current research was to show if there is a relationship between residence place and the selection of the three sets of skyline photos (Table 4). The study city consists of 8 districts, divided into traditional, contemporary and next-to-nature context. Districts 1, 2, 3 and 6 are areas with traditional context. Districts 5 and 7 are areas with a contemporary context. Districts 4 and 8 are suburban areas of the city and have next-to-nature context (Table 4).

**Table 4:** The chi-square test of the relationship between the district of residence and the selection of the photos.

District		The skylines from which photos were taken					Total
		Nature Set	Traditional Set		Contemporary Set		
1 (Traditional)		48 (84.2%)	9 (15.8%)		0		57
2 (Traditional)		33 (78.6%)	9 (21.4%)		0		42
3 (Traditional)		18 (75%)	6 (25%)		0		24
4 (Next to Nature)		30 (90.9%)	3 (9.1%)		0		33
5 (Contemporary)		78 (89.7%)	0 (0%)		9 (10.3%)		87
6 (Traditional)		51 (81%)	12 (19%)		0		63
7 (Contemporary)		12 (66.7%)	3 (16.7%)		3 (16.7%)		18
8 (Next to Nature)		21 (87.5%)	3 (12.5%)		0		24
Total		291 (83.6%)	45 (12.9%)		12 (3.4%)		348
N: 348	Pearson Chi-Square value: 50.203			df: 14		Asymp. Sig. (2-sided): 0.000	

The inferential test values show that the chi-square value is significant at a confidence level of 99.9% as the significance level is 0.000. Therefore, the null hypothesis indicating no association between the region and the selection of the photo sets was rejected and the main hypothesis confirmed, and it was specified that there is a relationship between the two. A review of Table 4 shows that some of the respondents residing in districts 5 and 7 selected the photos related to the contemporary context, while others did not select them. Table 4 shows that people living in the district 3 were highly willing to select the photos relevant to the traditional context (25%). District 2 also partly preferred the skyline photos of the traditional context. However, this percentage of the selection was lower in other districts. The main reason underlying the difference in the view of district 3 can be found in the traditional context of the people living in the district.

### 3.5 The relationship between the expressed reasons for selecting the photo and selecting the photo sets to hang on the wall

In one part of the questionnaire, the respondents were asked to select one photo out of three sets of the skyline photos to hang on the wall at the workplace or home and also expressing the reasons for their selection. In the frequency distribution table, the selection of the photo sets and the frequency of the stated reasons were reported. Then, all of the options related to the selection of the photos and the question asked on the reasons for selection were converted into artificial distance variables, and in case of selecting one set of the photos, the code became one and if not zero. In addition, in the case of selecting the reasons for the choice, the code became one, and if not zero. Therefore, the relationship between the selected sets of the photos was explained, and the stated reasons were tested using Pearson Correlation Test (Table 5).

**Table 5:** Pearson Correlation Test between the selection of the photos and the reasons for the selection to hang on the wall.

Variables		Distinctiveness	Excitement	Peacefulness	Attractiveness	Exhilarating	Simplicity	Originality	Memorability
Nature skyline	Correlation	-,431∗∗	,041∗	,482∗∗	-,188∗∗	,058∗	,041∗	,058	-,205∗∗
	Significance	,000	,438	,000	,000	,271	,438	,271	,000
Traditional Skyline	Correlation	,503∗∗	-,036∗	-,384∗∗	,041∗	-,051∗	-,036∗	-,051	,234∗∗
	Significance	,000	,496	,000	,436	,334	,496	,334	,000
Contemporary Skyline	Correlation	-,056∗	-,017	-,272∗∗	,312∗∗	-,024	-,017	-,024	-,017
	Significance	,289	-,748	,000	,000	,648	,748	,648	,748

∗ Correlation is significant at the 0.05 level.

∗∗ Correlation is significant at the 0.01 level.

### 3.6 Respondents' attitude towards photos based on the skyline theme

The attitude of the respondents during showing different sets of photos based on the skyline theme and evaluating their opinions has been measured as a 7-point differential scale. According to the scoring, the scale of this variable is from positive three to negative three and the average option has a neutral score of zero (Table 6).

**Table 6:** The averages of the characteristics of the 3 photo sets of nature, traditional and contemporary skylines.

	Contemporary Skyline	Traditional skyline	Nature skyline
Artificial-Natural	1.92	-0.38	-2.48
Rectilinear-Curvilinear	1.62	0.37	-0.92
Closed-Open	1.73	0.4	-1.5
Simple-Complex	-1.08	0.45	0.65
Abrupt-Smooth Transitions	1.2	-0.36	-0.96
Chaotic-Orderly	0.88	-0.07	-0.58
Regular-Irregular	-0.06	0.37	0.28

## 4 Discussion

Results (Table 5) show an inverse and significant relationship between the selection of the nature skyline photos and the reasons including memorability and attractiveness, since those who had such reasons in mind did not choose the nature skyline photos. In the meantime, there was a direct relationship between the selection of these photos and the peacefulness factor. Thus, by selecting the photos of this set, the peacefulness factor was expressed as the primary reason. In addition, the relationship between the selection of the nature skyline photos and the reasons including excitement, exhilaration, simplicity, and originality was positive, yet not significant, and fundamentally these reasons have no relationship with the selection of the first photo set. Regarding the selection of the skyline photos of the traditional context and the stated reasons for the choice, the results indicate that there is a positive and significant relationship between the selection of the reasons (distinctiveness and memorability) and the selection of the skyline photos of the traditional context. In the meantime, there is an inverse relationship between the choice of the skyline photos of the traditional context with the choice of the reason namely peacefulness. Thus, those who sought for peacefulness in the photos did not select skyline photos of the traditional context. In other cases, the selection of the skyline photos of the traditional context did not have any relationship with the reasons for choosing the other remaining reasons, and the relationships were not significant despite their negativity.

The relationship between the stated reasons and the selected photos of the skyline photos of the contemporary context indicates that the only reason for such selecting was attractiveness. In contrast, those who did select this photo set did not select the reason namely peacefulness. The results also showed that the reasons for dissatisfaction with contemporary skyline include Artificial, Closed, Rectilinear, Abrupt Transitions, Complex and Irregular, respectively. Based on the results obtained from the difference between the real averages of the seven features of skylines (Table 6), people who have chosen the contemporary skyline set of photos are more likely to think that this set of photos has features such as natural, open, smooth transitions and regular, as well as slight rectilinear; while the other group who chose the other photo sets were mostly of the opinion that the photos of the contemporary skyline set are strongly artificial, severely rectilinear, completely close, and many abrupt transitions, and relatively irregular.

**Figure 5:**
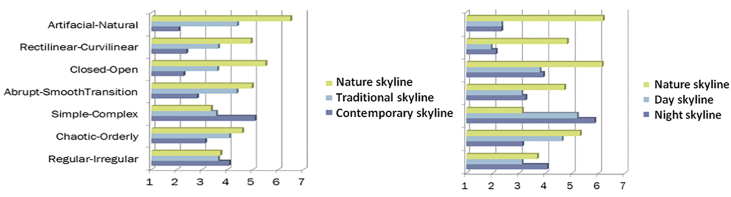
Comparison of the results of this research with Nasar and Terzano (2010). The chart on the left shows the results of this research and the one on the right shows the results of Nasar and Terzano 's research.

Also, based on the results of this study and in comparison with Nasar and Terzano (2010) study, it was found that the characteristics of the nature skyline, which was common in both studies, are very similar (Figure 5), and in other words, in this case, the results of this The research confirms the results of Nasar and Terzano's research. That shows that respondents in different cultures have similar judgments about this issue.

In the study, it was tried to select the photos based on the evaluation by the eyes. We reviewed about 2,000 photos of the city for the final selection of photos. These photos were taken from different angles and in different frames of the city. However, there are other methods for choosing photos based on computer analysis to increase the accuracy in selecting and examining their features in more detail (see Naik et al., 2017). It should also be noted that the findings and the type of skyline examined in this study are based on the specific features of a city in Iran; therefore to generalize the results, it is necessary to conduct similar research in other cities. In order to the ease of the respondents, ordinal variables were used. Since the level of independent and dependent variables was ordinal, it was not possible to apply regression analysis. If regression analysis was possible, the results could be more accurate and could clarify the dimensions of the problem.

## 5 Conclusion

The main question of the research was what type of skyline is preferred by people and what the reasons for this preference are. The research also attempted to explore the impacts of tall buildings in contemporary architecture, the city skyline, and the citizens' preference for the city skyline. It should be mentioned that in this study buildings taller than 23 m, which means buildings taller than a 7-story building, are considered tall buildings, which can be different in other cities. Besides, the research sought to know whether people prefer the existing alterations or not and whether these alterations have been merely imposed on people's preferences and tastes by urban architects and designers. Then the participants were asked to answer some questions, and it was found out that they prefer the nature skyline and that of the traditional context over the skyline of the contemporary context, and they consider peacefulness, memorability, and distinctiveness as their reasons for the selection. On the other hand, the research indicated that the respondents' residence place can influence attitude to prefer the skyline, in a fashion that the people living in the contemporary context of the city did mostly select the skyline of the contemporary context as their favorite skyline, and they did not look for the sense of peacefulness in the skyline, rather they stated attractiveness as the reason for the selection. And those living in the contemporary districts and apartments sought for peacefulness in other parts of the city. In addition, the investigations indicate that the two variables of age and gender have no effect impact on the citizens' preference for the favorite urban skyline. However, it was shown that a higher level of education leads to a higher tendency to select the photo set skyline of the traditional context and contemporary context. Physical features measured in this study are only a part of the features of aesthetics. It is suggested that in future research, different dimensions of aesthetics, including symbolic aesthetics, be examined, which is ignored in this study because of the breadth of topics. Also, in the dimensions of sensory and formal aesthetics that were specifically studied in this research, there are a lot of issues for research to achieve other aesthetic criteria such as fit and balance, which can shed more light on the relationship between aesthetics and skyline.

## Declarations

### Author contribution statement

Mehrdad Karimimoshaver: Conceived and designed the experiments; Performed the experiments; Analyzed and interpreted the data; Contributed reagents, materials, analysis tools or data; Wrote the paper.

Mastooreh Parsamanesh: Performed the experiments.

Amir Mosavi, Farshid Aram: Contributed reagents, materials, analysis tools or data.

### Funding statement

This research did not receive any specific grant from funding agencies in the public, commercial, or not-for-profit sectors.

### Data availability statement

Data will be made available on request.

### Declaration of interests statement

The authors declare no conflict of interest.

### Additional information

No additional information is available for this paper.
